# Synchronization of Firing in Cortical Fast-Spiking Interneurons at Gamma Frequencies: A Phase-Resetting Analysis

**DOI:** 10.1371/journal.pcbi.1000951

**Published:** 2010-09-30

**Authors:** Nathan W. Gouwens, Hugo Zeberg, Kunichika Tsumoto, Takashi Tateno, Kazuyuki Aihara, Hugh P. C. Robinson

**Affiliations:** 1 Department of Physiology, Development and Neuroscience, University of Cambridge, Cambridge, United Kingdom; 2 Department of Neurobiology, Harvard Medical School, Boston, Massachusetts, United States of America; 3 Nobel Institute for Neurophysiology, Department of Neuroscience, Karolinska Institutet, Stockholm, Sweden; 4 Aihara Complexity Modelling Project, ERATO, Japan Science and Technology Agency, Tokyo, Japan; 5 Institute of Industrial Science, The University of Tokyo, Tokyo, Japan; 6 Graduate School of Engineering Science, Osaka University, Osaka, Japan; 7 PRESTO, Japan Science and Technology Agency, Saitama, Japan; Université Paris Descartes, Centre National de la Recherche Scientifique, France

## Abstract

Fast-spiking (FS) cells in the neocortex are interconnected both by inhibitory chemical synapses and by electrical synapses, or gap-junctions. Synchronized firing of FS neurons is important in the generation of gamma oscillations, at frequencies between 30 and 80 Hz. To understand how these synaptic interactions control synchronization, artificial synaptic conductances were injected in FS cells, and the synaptic phase-resetting function (SPRF), describing how the compound synaptic input perturbs the phase of gamma-frequency spiking as a function of the phase at which it is applied, was measured. GABAergic and gap junctional conductances made distinct contributions to the SPRF, which had a surprisingly simple piecewise linear form, with a sharp midcycle break between phase delay and advance. Analysis of the SPRF showed how the intrinsic biophysical properties of FS neurons and their interconnections allow entrainment of firing over a wide gamma frequency band, whose upper and lower frequency limits are controlled by electrical synapses and GABAergic inhibition respectively.

## Author Summary

Oscillations of the electrical field in the brain at 30–80 Hz (gamma oscillations) reflect coordinated firing of neurons during cognitive, sensory, and motor activity, and are thought to be a key phenomenon in the organization of neural processing in the cortex. Synchronous firing of a particular type of neuron, the inhibitory fast-spiking (FS) cell, imposes the gamma rhythm on other cells in the network. FS cells are highly interconnected by both gap junctions and chemical inhibition. In this study, we probed FS cells with a synthetic conductance stimulus which mimics the electrical effect of these complex connections in a controlled way, and directly measured how the timing of their firing should be affected by nearby FS neighbours. We were able to fit a mathematically simple but accurate model to these measurements, the “synaptic phase-resetting function”, which predicts how FS neurons synchronize at different frequencies, noise levels, and synaptic connection strengths. This model gives us deeper insight into how the FS cells synchronize so effectively at gamma oscillations, and will be a building-block in large-scale simulations of the FS cell network aimed at understanding the onset and stability of patterns of gamma oscillation in the cortex.

## Introduction

Rhythmic oscillations of concerted electrical activity can occur in the neocortex and hippocampus at gamma frequencies (30–80 Hz), and are thought to be associated with a variety of cognitive tasks including sensory processing, motor control, and feature binding [1], [2]. A striking feature of gamma oscillations is their ability to be generated locally in the neocortex. Local gamma oscillations can be produced by pharmacological [3], [4], electrical [5] or optogenetic [6] stimulation. *In vivo*, synchronous gamma oscillations may be highly localized or widely distributed, even between hemispheres, with or without phase lags between different areas and layers [1]. It appears, therefore, that local neocortical circuits have an intrinsic capability for generating gamma oscillations, while sensory inputs and connections from other brain regions may shape the complex spatial patterns of oscillatory interaction.

Synchronized firing of cortical inhibitory interneurons has been implicated in the production of these rhythms in many experimental and modeling studies. During spontaneous network activity of the neocortex *in vivo*, the power of intracellular voltage fluctuations at frequencies higher than 10 Hz is dominated by inhibitory postsynaptic potentials, which are correlated with the extracellular gamma rhythm, and which synchronously inhibit nearby pyramidal cells [7]. A recent study using conductance injection in neocortical pyramidal cells indicated that gamma-frequency-modulation of firing is almost completely determined by their inhibitory input [8]. In the hippocampus and cortex, models of interneuron activity suggest that network oscillations depend on mutually inhibitory synaptic conductances [9], [10], [11].

Fast-spiking (FS) inhibitory interneurons are coupled by electrical synapses in addition to mutual and autaptic inhibitory synapses [12], [13], [14], [15]. Electrical synapses alone [12], [13] or in combination with GABAergic synapses [14] can produce synchronous firing in pairs of these interneurons *in vitro*. In addition, the biophysical properties of FS neurons appear to be ideally suited to generating gamma rhythms: they have a hard (“type 2”) onset of regular firing at about 30 Hz [16], which means that they can be easily entrained at this frequency. They also show a strong intrinsic drive for spike generation at gamma frequencies when stimulated with broadbrand conductance noise [17]. Recently, selective optical stimulation of FS interneurons, but not of pyramidal neurons, was shown to cause gamma oscillations [6]. Electrical synapses amongst mutually inhibitory interneurons have been found to increase the precision of synchrony in simulation studies [18], [19], [20]. However, the relative roles of chemical inhibition and gap-junctional coupling in shaping synchronous oscillations in the cortex are still unclear.

The theory of synchronization of coupled oscillators uses the concept of phase dynamics to evaluate the stability of the relative phase of coupled oscillators in time [21], [22]. The key to this approach is to determine the effect of a very small perturbing input on the phase of oscillation (“phase resetting”), as a function of the point in the oscillation cycle at which it occurs. This is most often used, under the assumptions of weak coupling and linear summation of phase shifts, to account for how the relative phase of presynaptic and postsynaptic cells evolves from cycle to cycle.

However, as described above, FS cells in the cortex are actually coupled quite strongly to other FS neighbours, with large postsynaptic conductance changes caused by each presynaptic action potential. Here, we have used synthetic conductance injection, or dynamic clamp, to directly measure the phase-resetting response to conductance inputs mimicking the effects of presynaptic action potentials, while systematically varying the relative strengths of electrical and GABAergic inhibitory conductances. The compound synaptic connections between FS neurons, together with the intrinsic spike-generating properties of FS neurons, give rise to a distinctively-shaped phase-resetting relationship, or “synaptic phase-resetting function”, which ensures rapid and precise synchronization over a large gamma-frequency range.

## Results

### Conductance injection reproducing synaptic input

**Figure 1:**
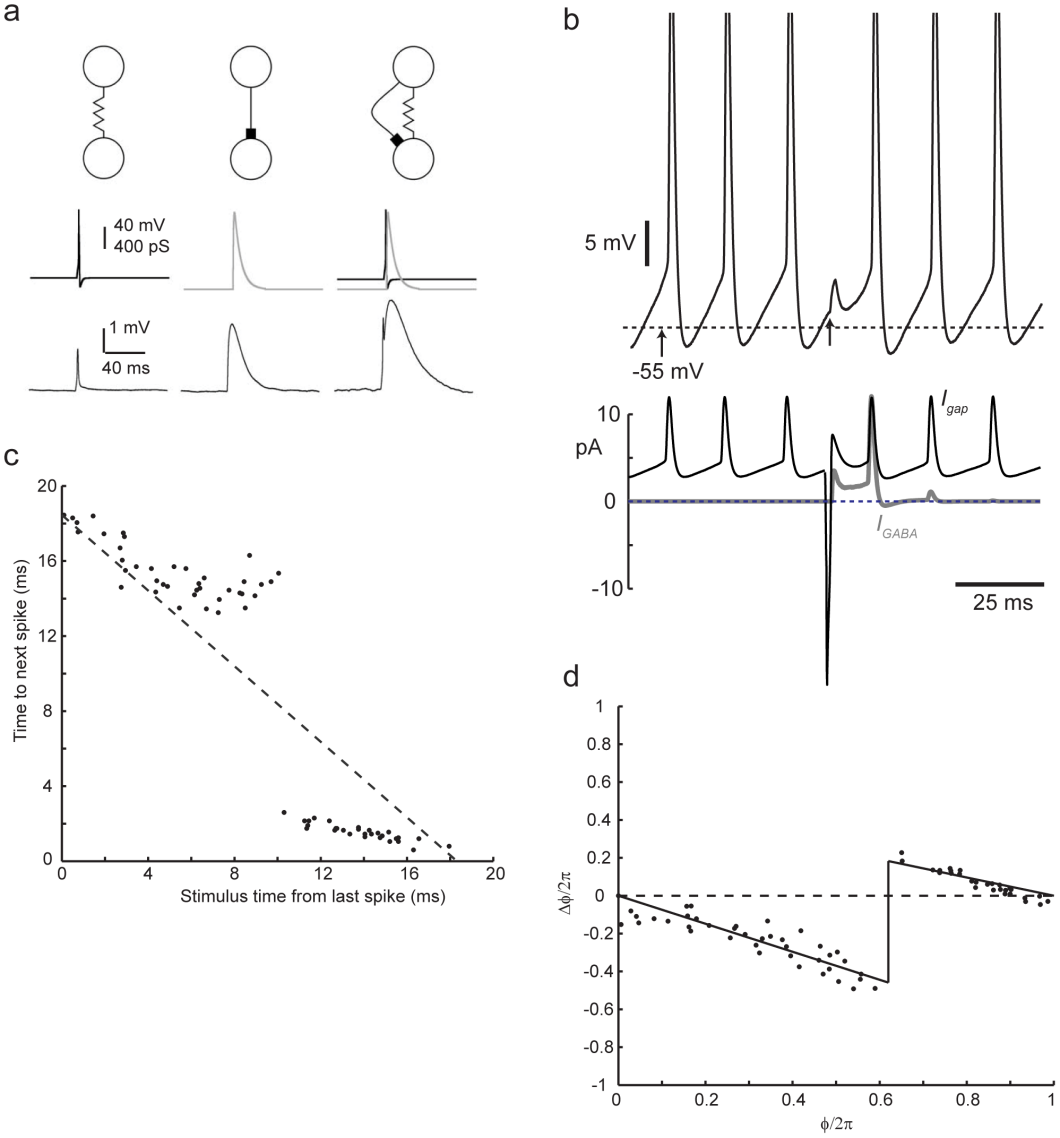
Synaptic connections between FS cells simulated by conductance injection. a) *Left*, an electrical synapse (*top*), simulated by a time-varying *E*_rev_ signal (*middle*), and a constant conductance of 750 pS, produces a spikelet in the recorded neuron (*bottom*). *Center*, a GABAergic synapse (*top*). A transient of conductance reversing at −55 mV mimics a GABA_A_ synaptic input (*middle*), producing a small depolarization from rest (*bottom*). *Right*, a compound electrical/GABAergic connection (top). Combined input from both types of conductances (*middle*) produced a response with a sharp, electrical synaptic component followed by a longer-lasting IPSP (*bottom*). Each panel is recorded from a different cell. b) expanded view of the membrane potential trajectory (top, spike peaks truncated) and injected currents (bottom, gap-junctional current in black, current through GABAergic conductance in gray, outward current is represented upwards) during application of a single compound conductance perturbation (*g_e_* = 0.2 nS, *g_i_* = 1.4 nS) starting at the time indicated by an arrow, in this case inducing a delay in the subsequent spike time. c) Relationship between time at which input is applied and the time to next spike and d) corresponding phase-resetting relationship, or synaptic phase-resetting function.

FS cells in rat somatosensory cortical slices were identified by their morphology, action potential shape and characteristic firing pattern in response to depolarizing current injection [12], [13], [23], [24]. FS cells fired high frequency, nonadapting trains of action potentials during depolarizing current steps, occasionally interrupted by pauses with subthreshold oscillations, particularly around threshold [16] (see Methods). We used conductance injection/dynamic clamp [25], [26] to reproduce the effects of electrical and chemical synapses (Fig. 1, see Methods). In FS cells, both gap junctions and GABAergic synapses from neighboring cells are located perisomatically [14], so that point conductance injection at the soma should reasonably reproduce the electrical effects of synaptic inputs. Gap junctions were implemented as a static conductance between the recorded cell and a “voltage-clamped” trajectory of “presynaptic” membrane potential. This “voltage-source” approximation, importantly, allowed us to characterize a functional mapping between the presynaptic spike time and the influence on postsynaptic membrane potential, without considering any reverse effect of gap-junctional current on the presynaptic cell. This is valid as long as the presynaptic cell is considered to be much more strongly controlled by its other inputs, as when it is already part of a synchronous assembly (see Discussion). It is estimated that each FS cell is gap-junction coupled, directly or indirectly, with a measurable coupling, to between 20 and 50 other FS neurons [27], so that if the presynaptic cell is quite strongly-driven by a major proportion of these inputs, then the effect of any one can be neglected. At rest, this gap-junctional input produced a small postsynaptic spikelet (Fig. 1a, left), very similar in size and shape to those observed with natural electrotonic coupling [12], [13]. We also measured coupling coefficients (the ratio of postsynaptic to presynaptic potential change) for gap-junctional type conductance. These were similar to physiological values, and larger for step inputs (0.05–0.22) than for spike inputs (0.01–0.05), owing to low-pass filtering by the combined effects of gap junctional conductance and membrane resistance and capacitance [28].

Many pairs of FS cells are connected by both GABAergic (GABA_A_, chloride conductance) and electrical synapses [12], [13], [14]. We simulated GABAergic synaptic input using conductance injection (Fig. 1a, middle). The GABA reversal potential (E_GABA_) was set to −55 mV, based on gramicidin-perforated patch measurements in this cell type [10], [29], considerably more depolarized than in pyramidal neurons [30]. Thus, inhibition is shunting in the range of membrane potentials between spikes during repetitive firing (Fig. 1b). Starting from the resting potential, the “IPSP” is a small depolarisation lasting about 40 ms, again very similar to natural IPSPs in these cells. At the resting potential, a stimulus with both electrical and GABAergic components produces a biphasic depolarizing response (Fig. 1a, right) with the gap-junctional potential visible just before the larger GABAergic potential. Unlike the gap-junctional spikelet, though, the amplitude of the GABAergic potential can change sign in the subthreshold, interspike range of membrane potentials, reversing around E_GABA_ [12].

### Perturbing spike timing

To determine how this compound synaptic input shifts the timing of periodic firing in an FS cell, we applied conductance inputs during periodic firing elicited by a maintained excitatory stimulus, a step of excitatory conductance reversing at 0 mV. An example response to a compound “synaptic” perturbation is shown in Fig. 1b. In phase-resetting analysis of synchronization, the state of the neuron is characterized by a single quantity, the phase angle, 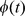, which – in the absence of any perturbations - increases linearly with time, and which is reset to zero whenever it reaches 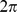, corresponding to the occurrence of a spike [21]. The variability of interspike intervals can be represented by adding additional noise, due to stochastic gating of ion channels and other intracellular sources of variability, to the rate of change of 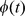. To measure the phase *resetting*, or shift in the phase, produced by synaptic-like conductance inputs, we applied isolated single inputs during long trains of periodic firing. Fig. 1c shows the relationship between the time *t_p_* at which an input (in this case a compound gap/GABA input) is applied, relative to the time of the preceding spike, and the time until the next spike occurs (*t_n_*). This clearly deviates from the line of slope −1 (dotted line) expected in the absence of any input, and has two approximately linear regions separated by a sharp transition. Note the characteristic progressive decrease in the variability of this relationship, as *t_p_* increases – this is because the earlier the input arrives, the more time is left for integrating the effects of noise before the next spike.

### The synaptic phase-resetting function and the effect of varying electrical and inhibitory conductances

**Figure 2:**
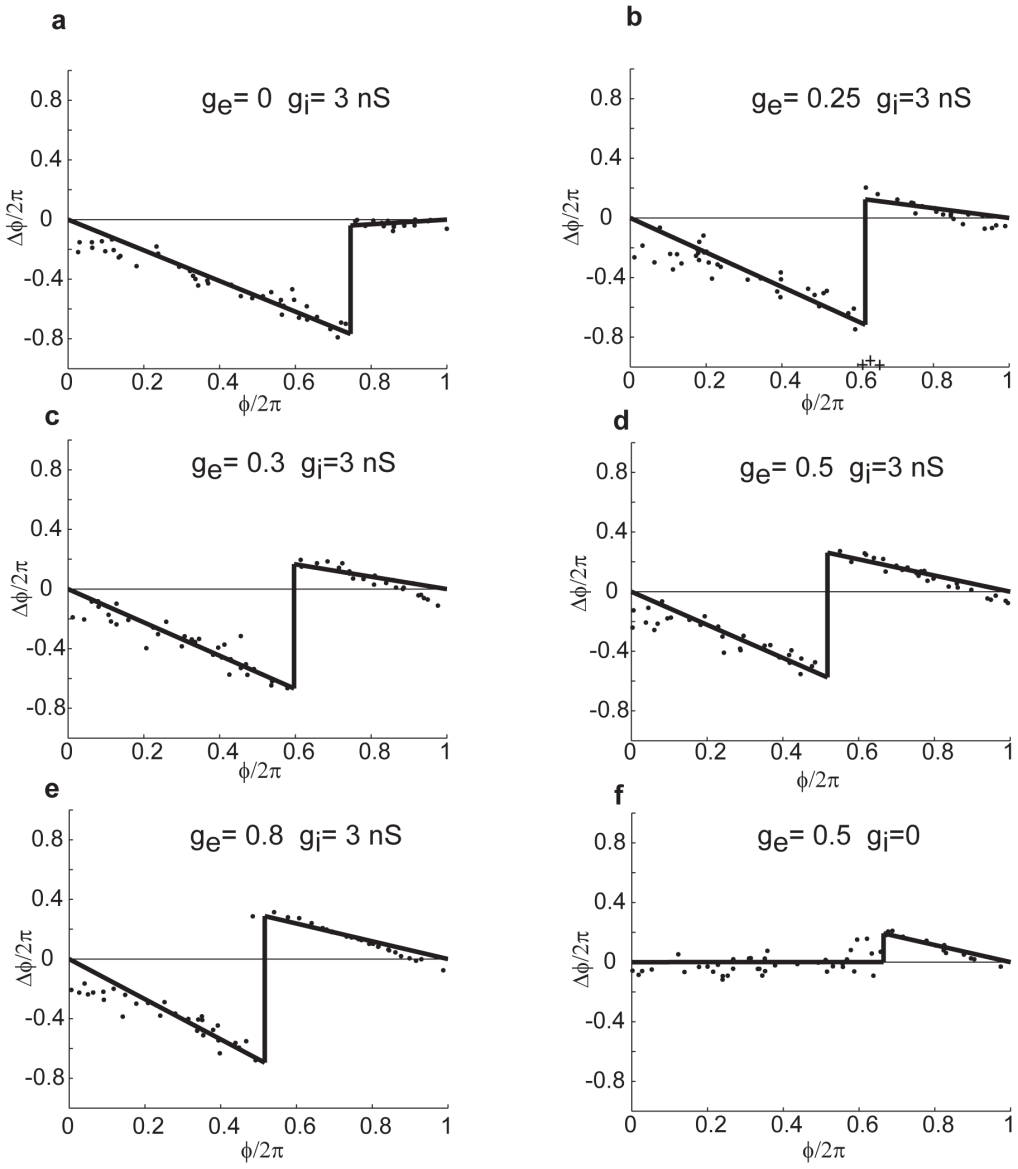
SPRFs in one cell for different strengths of gap-junctional and inhibitory conductance. a) inhibition only. Phase delay increases linearly as the phase of onset of the synaptic perturbation increases, before an abrupt loss of sensitivity late in the cycle. b) As gap-junctional conductance is introduced, phase delay switches to a region of linear phase advance late in the cycle “+” symbols indicate outliers excluded from the piecewise linear fit using Grubbs' test, as described in the Methods. c), d), e). As gap-junctional conductance is increased, the slope of the phase advance region becomes larger, and the point of switching is shifted progressively earlier in the cycle. f. switching off inhibition completely leaves only the late phase advance (compare to (d)).

From this relationship, we can estimate the phase at the moment that each input is applied, and the amount of phase resetting 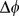 produced by the input (see Methods), as shown in Fig. 1d, in which 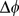 is plotted as a function of 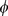. This relationship - the total phase-resetting effect of a synaptic input as a function of the phase at which it arrives – we will refer to as a synaptic phase-resetting function (SPRF), to distinguish it from a classical phase response or phase-resetting curve, which normally describes responses to very small, brief inputs, whose effects can be considered to sum linearly. We examined how the parameters of the synaptic input determine the shape of the SPRF, by varying the magnitude of gap-junctional and GABAergic conductance, applied individually or together (Fig. 2a–f). These components vary physiologically, since FS cells' interconnections can be purely GABAergic (one-way or reciprocal), purely gap-junctional or both [12], [13], [14]. In addition, there is a wide range of electrical synaptic strengths [28].

Purely GABA input produced a phase delay early in the cycle, which increased during the cycle until an abrupt critical point, beyond which it had no effect (Fig. 2a). Introducing a small (250 pS) gap junction, caused a linear region of phase advance (Fig. 2b), as in Fig. 1d, which had an abrupt onset at a phase of about 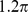. A sharp transition marks the boundary between this region and the first, phase delay part of the phase cycle. The slope of the phase advance region became more negative, and the boundary between the regions, designated the critical phase 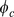, shifted earlier in the cycle, as gap junctional conductance increased (Fig. 2c, d, e). With no GABAergic input, a phase advance region produced by gap junctional input is seen in isolation (Fig. 2f).

Thus, GABAergic input retards, and gap-junctional input advances the phase of firing. For the compound gap/GABA input, the early region of phase delay has a slope determined by the amplitude of inhibition, *g_i_* (see Methods), and switches abruptly, midcycle, to a region of decreasing phase advance, whose slope is determined by *g_e_*, with no detectable sign of cancellation of the two regions in midcycle. The only clear interaction between the electrical and GABAergic components was that a larger gap junctional conductance shifted 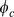 to earlier in the cycle.

To quantify the goodness of fit of the piecewise linear SPRF, we performed a chi-square test of 130 phase response curves (in total 6111 data points, 10 cells). For each SPRF, variance of phase was estimated from an unperturbed spike train within the same experiment (median 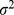 = 0.021 (rad/2π)^2^. 111 of 130 SPRFs contained no significant difference between the model fit and experimental result (*p*<0.05). The average reduced chi-square value was 0.80, meaning that the overall fit of the model is extremely good, given the measured degree of variance in the phase. On the whole, the relatively simple piecewise linear model performs remarkably well.

**Figure 3:**
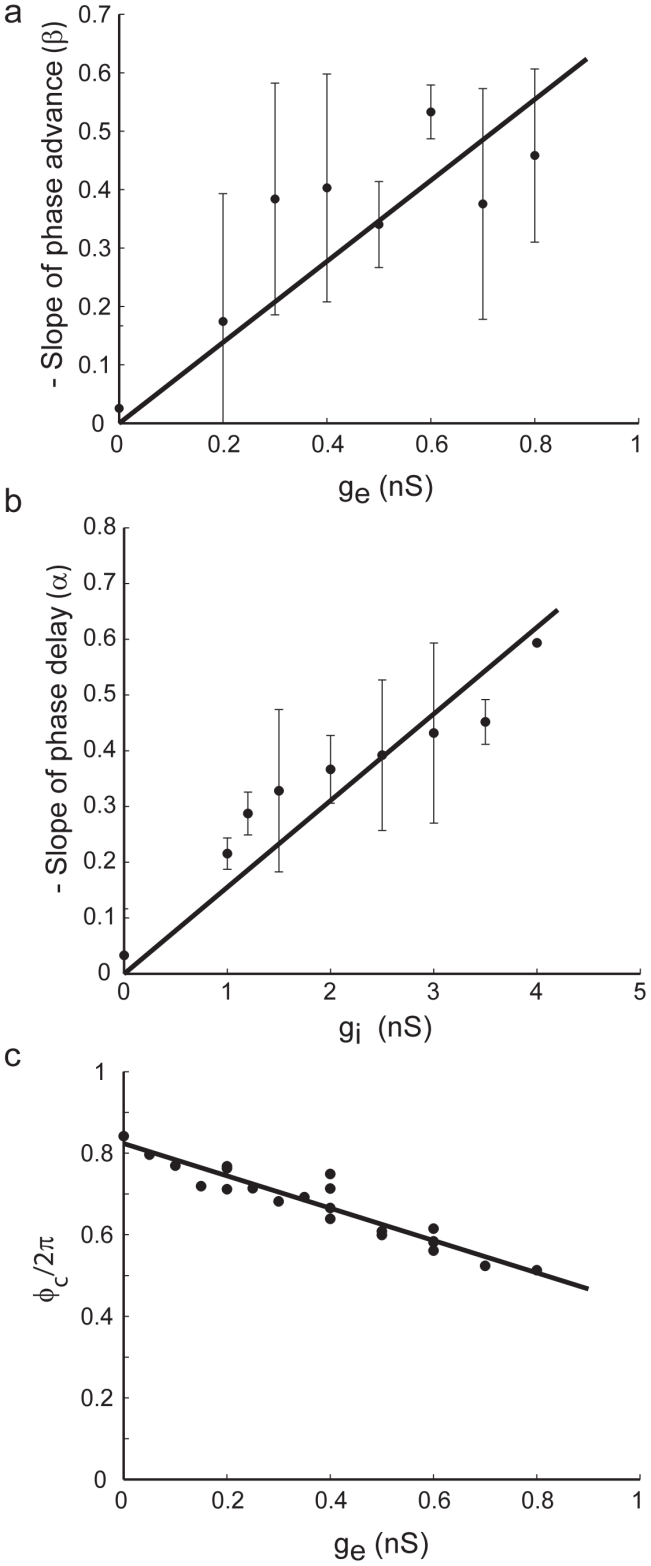
SPRF parameters depend on the strength of synaptic perturbation in a simple manner. a. Dependence of the phase advance slope (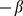) on the gap-junctional conductance (for 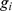 = 1.5 nS). Data pooled from 120 measurements in 10 cells. b. Dependence of the phase delay slope (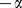) on 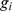. Data from 43 measurements in 7 cells. c. Dependence of the critical phase at which delay switches to advance (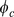) on the gap-junctional conductance 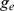 in one cell.

The dependencies of the slopes and breakpoint on the strengths of *g_i_* and *g_e_* were also fitted by linear relationships (Figure 3). The negative slope of the region of phase delay was proportional to inhibition (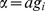, Fig. 3b), the negative slope of the phase advance region was proportional to excitation (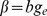, Fig. 3a), while 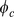 was weakly sensitive to *g_e_* (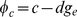, Fig. 3c). Average values of *a* and *b* of this piecewise linear model for the SPRF were *a* = 0.16/nS (n = 7 cells, 3 cells providing insufficient data for analyzing this dependency), b = 0.69/nS (n = 10 cells). *c* and *d* were more variable from cell to cell, and the pooled data in fact showed little overall dependence on *g_e_* (not shown). Nevertheless (e.g. Fig. 3c), the weak relationship is clear within individual cells.

### Entrainment by synaptic input

**Figure 4:**
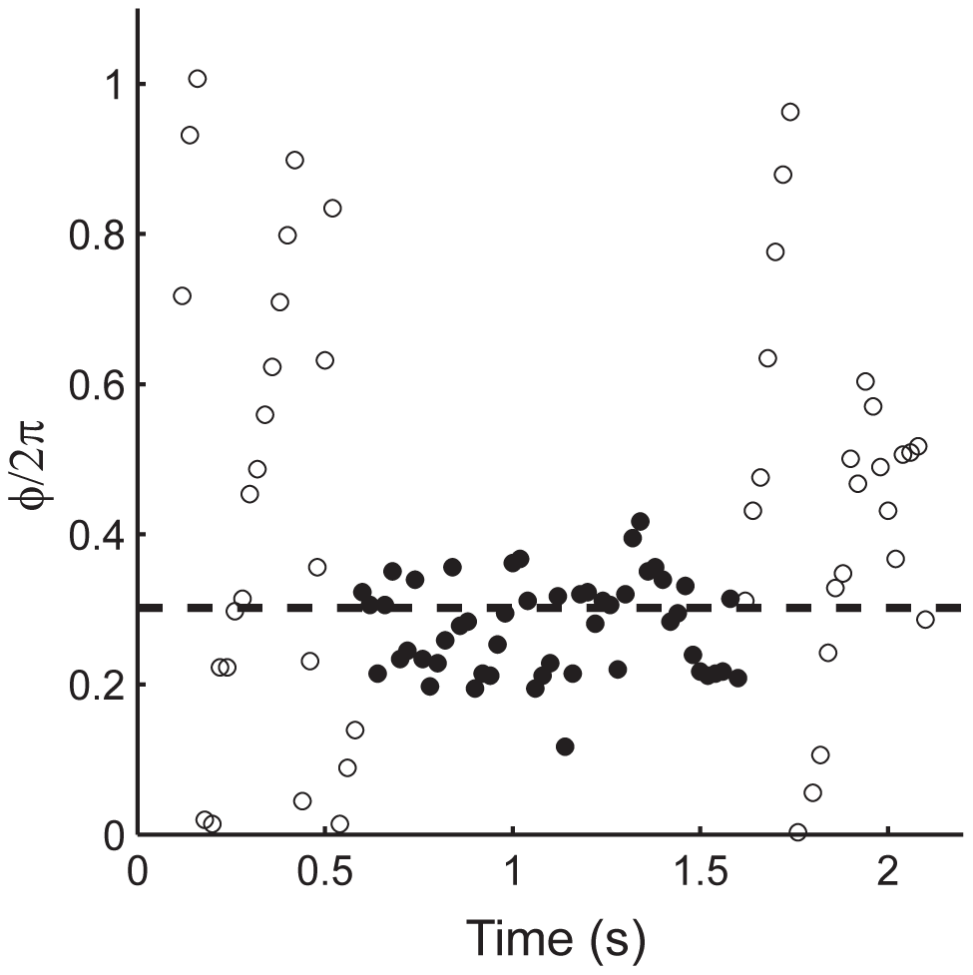
Entrainment of firing to a periodic conductance input. An example of a stroboscopic plot of the phase of a neuron, observed in phase with stimulation by a compound synaptic-like conductance of (*F* = 56 Hz, *f* = 50 Hz, *g_e_* = 750 pS and *g_i_* = 3 nS). The conductance pulses are applied during the period indicated by filled circles. Dashed line indicates the equilibrium solution of Eq. 2 for this cell.

Having established that conductances resembling the synaptic input of neighboring FS cells can consistently modify spike timing, we next tested the ability of FS cells to synchronize to, or to be entrained by this input. To visualize the time course of entrainment, we examined responses stroboscopically [22], sampling the phase of the FS cell at the times of periodic stimuli. Figure 4 shows such an experiment. Before the conductance pulses are switched on (*open circles*), the phase changes in a “sawtooth” pattern, reflecting detuning - the continuously growing phase difference between two oscillators of different frequencies. After the conductance transients begin (Fig. 4, *filled circles*), the phase quickly converges on a fixed value relative to the stimulus, at about 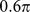 (dashed line), which matched the expected equilibrium phase difference from solving Equation 2 with parameters for this cell. Thus the FS cell becomes phase-locked and frequency-locked to the stimulus train, with spikes occurring around 0.6π before, or equivalently 1.4π after each stimulus. After the end of the stimulation train, the phase reverts to the drifting detuned state.

**Figure 5:**
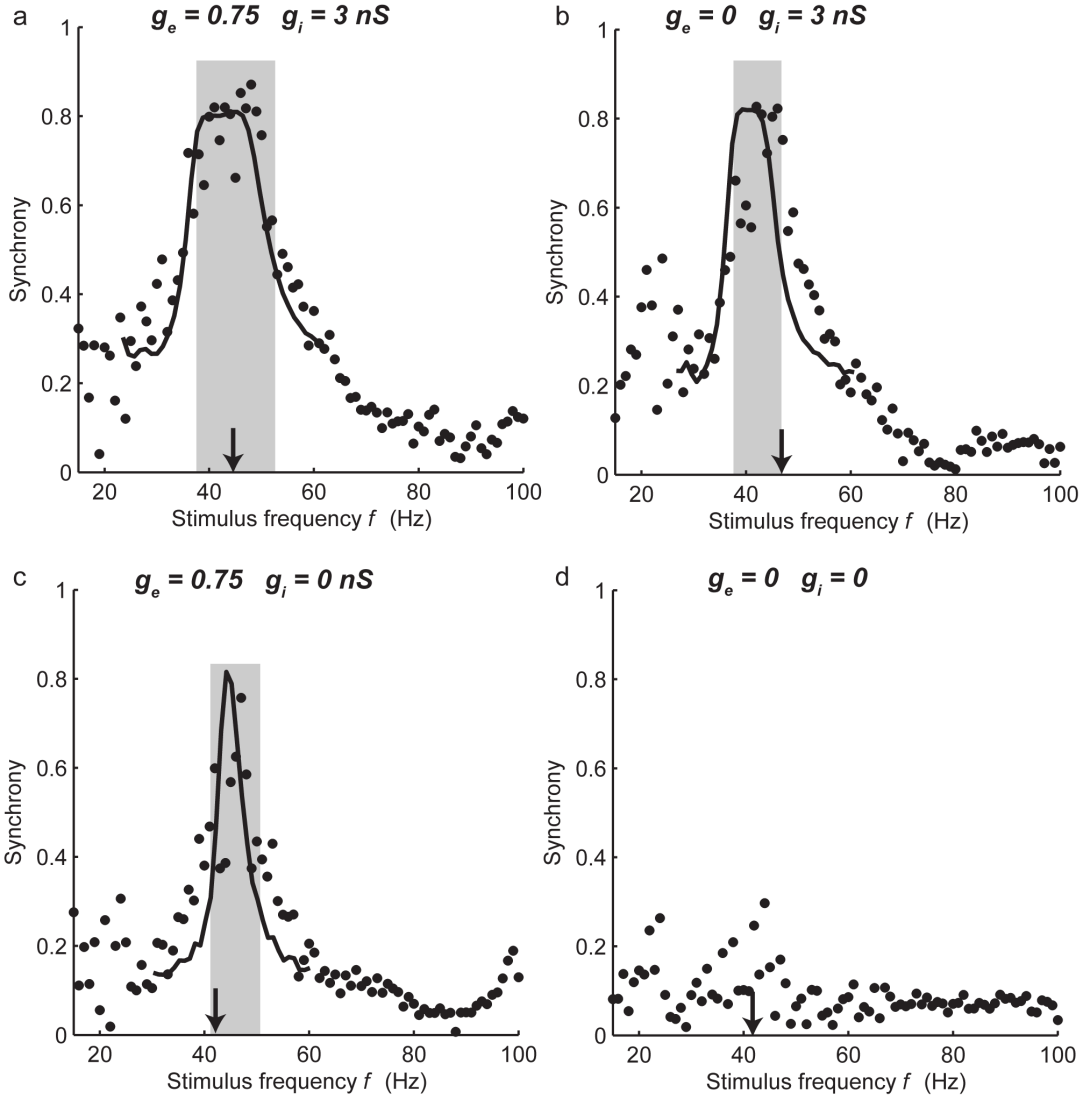
Frequency dependence of entrainment. The synchrony measure *S* (see Methods) is plotted as a function of the frequency of the entraining input. Conductance values as shown. a) compound gap-junctional/inhibitory input. b) pure inhibitory input. c) pure gap-junctional input. d) random level of synchrony in the absence of conductance input. Arrowheads indicate the natural firing frequency *F* in the absence of perturbations, and gray regions indicate the frequency bands of 1∶1 synchronization predicted by the measured SPRF. Solid curves in (a)–(c) show the calculated steady-state synchrony of the fitted noisy SPRF model.

The piecewise linear SPRF could also account for the frequency band over which synchronization was possible. Fig. 5 shows an experiment in which an FS neuron firing at a steady frequency *F* was stimulated repeatedly with a periodic synaptic conductance input at frequency *f*, and an index of the synchrony of the cell with the input (*S*, varying between 0 and 1, see Methods) was measured over a range of frequencies. As seen in Fig. 5a, this changes from a low level when *f* is very different from *F*, to a high value approaching 1, when 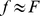. Because of the effects of noise in the neuron, there is no absolute phase locking (*S*<1), and the change in synchrony with input frequency does not have abrupt boundaries, but falls away continuously as the difference between *f* and *F* grows. It is clear that the central region of high synchrony lies below the unperturbed or natural firing frequency *F* when only inhibition is applied (Fig. 5b), above *F* when only gap-junctional conductance is applied (Fig. 5c), or both above and below *F* when a compound input is applied (Fig 5a). This observation was duplicated by the piecewise linear model of the SPRF, analysis of which (see Methods) predicted the 1∶1 synchronized frequency bands shown in gray, for the deterministic (noise-free) case – in this neuron, these boundaries corresponds to a synchrony of about 0.7. The synchronized frequency band is much narrower for either gap-junctional stimulation alone (Fig. 5b) or GABAergic inhibition alone (Fig. 5b). Iterations of the noisy stroboscopic map derived from the fitted SPRF (Eq. 2) showed that it could also reproduce the distribution of *S* adequately (black curves in Fig. 5a–c). Thus the piecewise linear model of the SPRF appears to account very well, both for the frequency range and degree of synchronization in noise.

### Frequency bands of deterministic and stochastic synchronization

**Figure 6:**
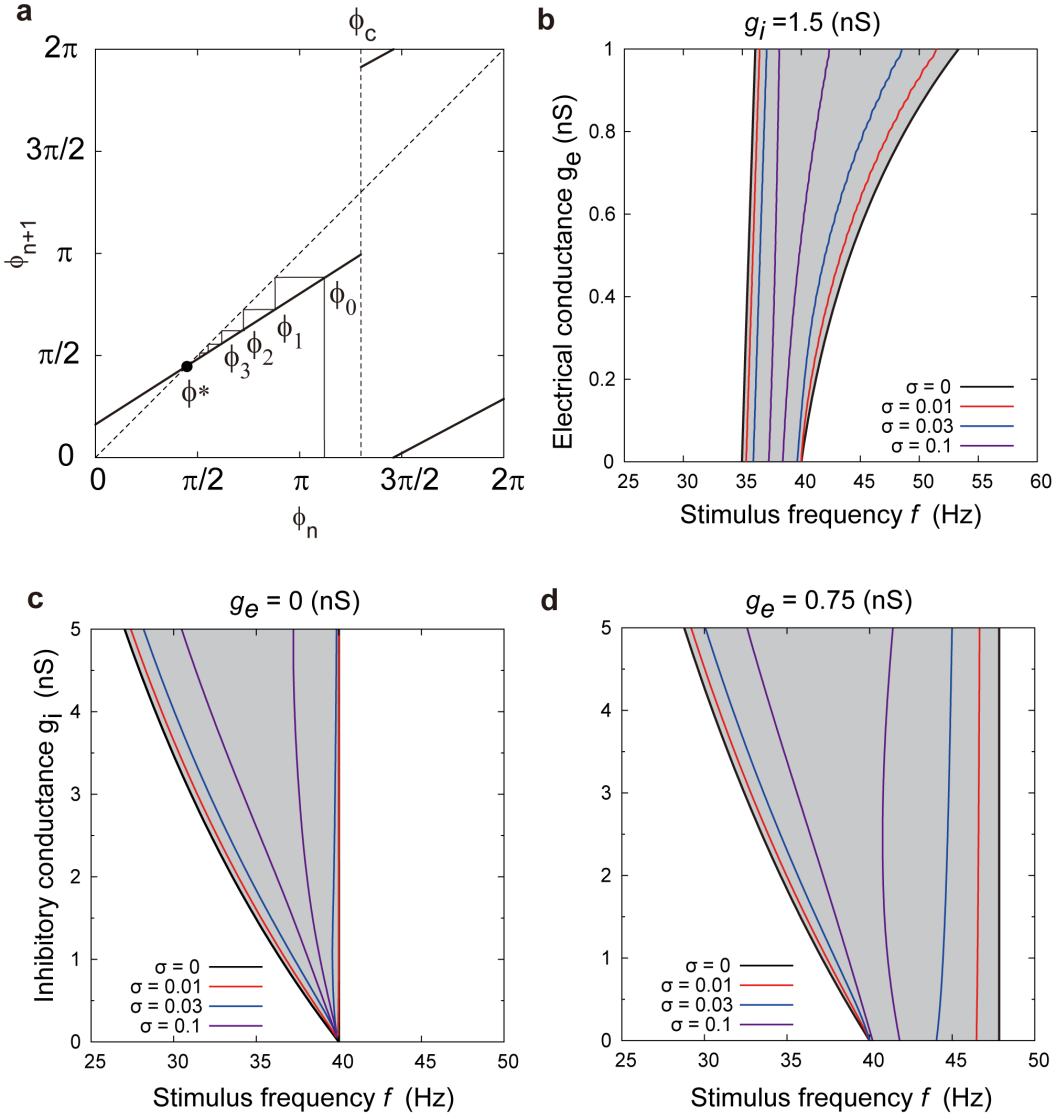
Bifurcation analysis of frequency bands of synchronization. a) piecewise linear map between phase at stimulus *n* and phase at stimulus *n+1*. The point ϕ* on the diagonal is a stable fixed point of the map, as illustrated by the converging orbit *ϕ*_1_, *ϕ*_2_, … showing that 1∶1 entrainment occurs at this stimulus frequency. b) bifurcation points of 1∶1 entrainment in the *g_e_*, *f* plane, *g_i_* = 1.5 nS. 1∶1 entrainment occurs in the gray regions. σ = 0, deterministic case. For σ>0, stochastic bifurcation points with added Gaussian noise in the phase (see text). c) synchronization region in the *g_e_*, *f* plane, with *g_e_* = 0. (d) as in (b), with *g_e_* = 0.75 nS. Raising *g_e_* strongly increases the upper frequency limit of entrainment, and weakly increases the lower limit. Noise shrinks the stochastic synchronization region. Parameters: *a* = 0.12/nS, *b* = 0.625/nS, *c* = 0.8*2π rad, *d* = 0.2*2π rad/nS.

We next used the SPRF to predict the frequency ranges of entrainment for different strengths of inhibition and electrical coupling (Fig. 6), by analyzing the bifurcations at the onset of synchrony in the stroboscopic map of the phase, i.e. the map of the phase of the postsynaptic cell at successive presynaptic spike times in a regular train (see Methods, equation 2). For the deterministic (zero noise) case, 1∶1 entrainment corresponded to a stable fixed point of the map, labelled 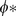 in the example shown in Fig. 6a. As the amount of detuning (difference between *f* and *F*) varies, the map shifts vertically, so that at certain stimulus frequencies, the fixed point disappears (at a “corner-collision” bifurcation [31]). Thus, it is possible to plot the regions in which there is synchronization in the 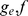 plane (Fig 6b) or the 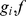 plane (Fig. 6c,d). These form Arnol'd tongues [22] in which the frequency range of entrainment shrinks as the synaptic strength is reduced.

This analysis shows a number of effects which are relevant to the physiological function of FS neurons. Increasing 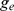 strongly increased the upper frequency limit of entrainment and weakly increased the lower limit (Figs. 6b). When 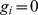 it is impossible to entrain firing with *f*<*F*. Conversely, with 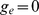, it is impossible to entrain for *f*>*F*, and increasing 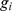 strongly reduces the lower frequency limit of entrainment (Fig. 6c,d).

Since physiologically, entrainment must occur in the face of considerable noise, we also investigated the effect of adding noise to the phase map. It is possible to define stochastic bifurcation points of the map *F*, at which there is a qualitative change in the nature of the stochastic dynamics. These points coincide with the deterministic bifurcation frequencies [32] for 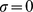 (see Methods for details). We examined the frequency extents of this kind of stochastic entrainment at different noise levels (Fig. 6b–d). In all cases, increasing the noise in the phase shrinks the region of entrainment. For 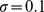 rad/2π, which was a typical noise level in these cells *in vitro*, the area of stochastic entrainment shrank to a third or less of the noise-free case. This noise-induced distortion is not symmetrical in the frequency axis. For example, Fig. 6d shows that in the absence of electrical coupling, the lower frequency limit of entrainment was highly susceptible to noise while the upper limit was not. The greater the level of electrical coupling (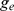), the more the upper limit was reduced by noise.

The SPRF makes several predictions. First, FS cells receiving purely electrical synaptic input will synchronize effectively when driven at frequencies higher than *F*. Higher frequencies can be followed with stronger electrical input. Second, cells will synchronize to purely inhibitory input at frequencies lower than *F*, and stronger inhibition allows lower frequencies to be followed. Third, combined electrical and inhibitory input allows cells to synchronize to frequencies both above and below their unperturbed frequency. Although noise diminishes the frequency band of synchronization, sometimes asymmetrically, these conclusions remain valid in the presence of noise. For typical strengths of combined electrical-inhibitory synaptic connections, 20 Hz or greater bandwidths of stochastic synchronization persist even in quite high levels of noise (σ = 0.1).

## Discussion

### Measuring the effect of synaptic conductance on phase of periodic firing

A number of previous theoretical and experimental studies have examined the phase-resetting properties of cortical neurons. Ermentrout and Kopell developed a theoretical approach to calculate what they termed the “synaptic interaction function” based on phase response curves and the assumption of weak coupling [33]. Reyes and Fetz (1993) stimulated synaptic inputs to regularly-firing pyramidal neurons to measure the phase resetting produced by EPSPs [34], while Stoop et al. (2000) used similar measurements to predict input frequency regions for entrainment and chaos [35]. Netoff et al. used dynamic-clamp to measure phase-resetting (or spike-time response curves) by artificial excitatory or inhibitory conductances in excitatory stellate cells of medial entorhinal cortex, and oriens-lacunosum-molecular interneurons in the CA1 region of hippocampus [36], and were able to demonstrate synchronization in pairs of neurons connected by artificial conductances mimicking synaptic connections, or between biological neurons and simulated neurons. In fast-spiking inhibitory cells, Mancilla et al. (2007) measured phase-resetting relationships for small current pulses (weak coupling) and showed that they could account quite well for synchronization of pairs of gap-junction coupled FS cells, both experimentally and in a biophysical model of FS neurons [37]. In this paper, we go further, by using conductance injection (dynamic clamp) to reproduce the combined effect of gap-junctional and strong synaptic connections, and using this to predict the resulting synchronized frequency bands, and their dependence on synaptic strength, including the effect of noise in the synaptic phase-resetting function on synchronization.

The conductance pulses which we have used are based on the physiological properties of the synaptic connections between FS neurons. In FS neurons of a basket morphology, APs initiate in the axon [38] arising usually from a proximal dendrite, [39] and receive many of their inhibitory connections and gap junctions from other fast-spiking interneurons perisomatically [14]. Thus, dynamic clamp recordings at the soma should provide a reasonably realistic simulation of the natural gap-junctional and fast inhibitory input.

In order to carry out this analysis, we have made the approximation that, between spikes, the presynaptic voltage of the gap-junctional input was held at a resting potential of −70 mV, . In other words, we have focused on the effect of gap-junctional current flow associated with the discrete event of the presynaptic spike. This approach does not take account of the way in which presynaptic membrane potential would gradually depolarize between spikes, if firing periodically. We have also ignored the two-way nature of coupling between cell pairs. In other words we model entrainment of one cell by another, rather than synchronization of a symmetrical coupled pair. Although both electrical and inhibitory coupling can often be asymmetrical [13], [40], they may also be quite symmetrical. However, the entrainment studied here models the situation where the presynaptic cell is already imperturbably-driven as part of a strong synchronously-firing assembly of FS neurons, so that the phase and frequency of its firing will be clamped to that of its predominant input. Thus, the SPRF that we measure should be an effective model for describing recruitment of new cells to such a synchronous assembly.

It is expected that the preferred firing frequency *F* of the postsynaptic cell may also affect the form of the SPRF, since the timing of intrinsic ion channel kinetics will shift relative to phase as the cycle length changes. In a few experiments where we were able to address this issue, we indeed found evidence of a change in the parameters of the SPRF model. *a*, the dependence of phase delay on *g_i_*, increased quite strongly as firing frequency increased, and 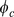 shifted earlier in the cycle as firing frequency increased. The dependence of *b* and *d* on firing frequency was not marked. The relatively strong effect on *a* may partly reflect the long duration of the IPSP conductance relative to the period of the cycle.

### The synaptic phase-resetting function

**Figure 7:**
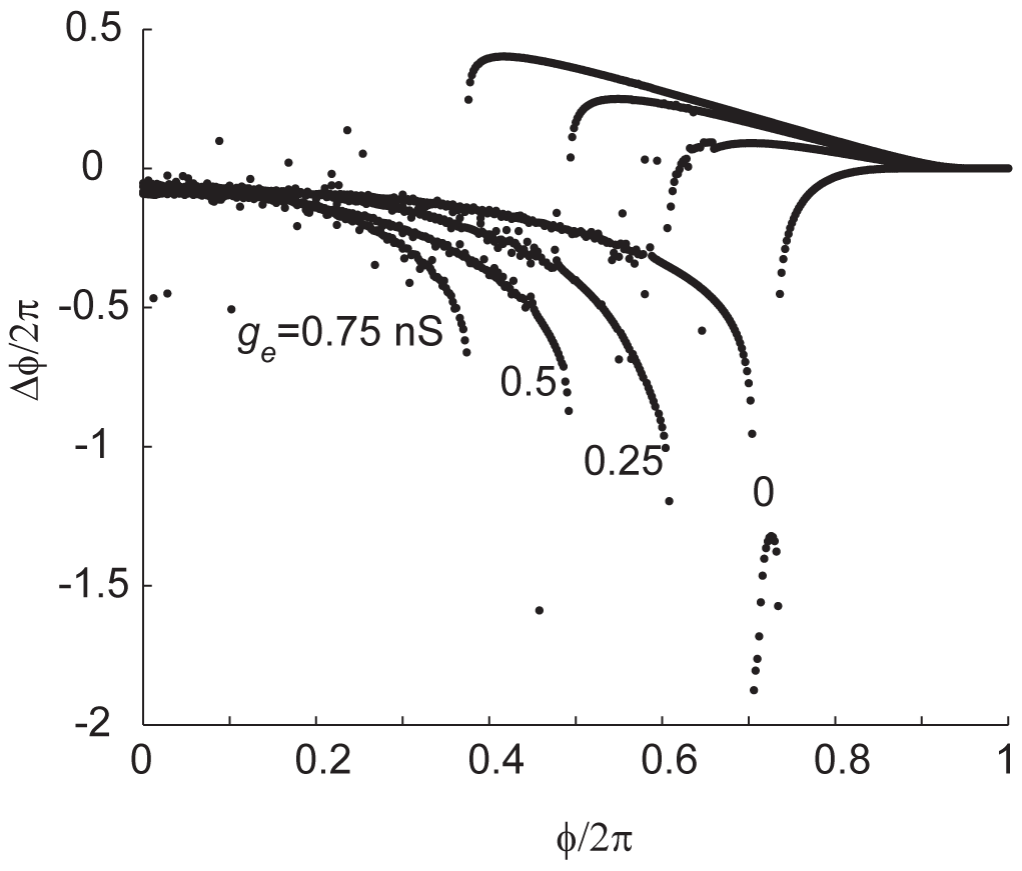
SPRF simulated for the Erisir *et al.*, 1999, fully-deterministic biophysical model of an FS cell. *g_i_* = 1.5 nS. 4 different values of *g_e_* are used as indicated. *F* = 40 Hz. Phase shifts are evaluated in steps of 0.002/2π in the onset phase of the compound synaptic input. Extraneous points lying off the main curves, particular for phase delays, reflect a complex local fine structure of the phase shift, around the central relationship.

The synaptic phase-resetting function, or SPRF, for compound input was distinguished by the following features: an extremely abrupt midcycle switch from phase delay to phase advance, which shifted weakly towards the early part of the cycle as the strength of electrical coupling was increased; amplification of the phase delay region by increasing inhibition; and amplification of the phase advance region by increasing gap-junctional coupling. We found that these qualitative features were also present in a biophysical model of firing in fast-spiking cells [41] (see Methods), incorporating voltage-gated sodium, Kv1.3 and Kv3.1/3.2 potassium channels, and stimulated with exactly the same inputs as used experimentally (Fig. 7). In this fully-deterministic model, we also observed a very fine local structure of fluctuations around the main relationship, particularly in the phase delay. Despite these qualitative similarities between the model and experimental results, there were also major differences. In experiments, phase advance was produced exclusively by gap-junctional conductance and phase delay exclusively by inhibition, while in the model, gap-junctional input did affect phase delay strongly early in the cycle – this was never observed experimentally. This deficiency of the biophysical model suggests that additional conductances expressed in FS neurons somehow help to confer a complete immunity to gap-junctional stimulation in the early, phase-delay part of the cycle. We surmise that the voltage-gated potassium conductance in this part of the cycle may actually be much higher than in the model, and that this may allow phase delay and advance to be regulated completely independently. Also, because of their relative timing, the effect of inhibition will outlast that of the gap-junctional current transient – thus phase delays caused by inhibition starting early in the cycle may in fact be caused more by their persistence until later in the cycle. In addition, the model shows a pronounced curvature in the phase delay region of the SPRF which was not noticeable in any experimental recordings. This might reflect the presence of other voltage-dependent conductances in real FS cells which effectively linearize this part of the relationship.

The sharp discontinuity between phase delay and advance which emerges at high synaptic strengths is a result of the particular intrinsic biophysical properties and the nature of the synaptic perturbation. It appears to be related to the “class 2” nature of the FS neuron threshold [16], and may be sensitively determined by the potassium conductance densities and kinetics [42], [43]. It was not observed for example in a class 1 excitable Morris-Lecar model. The discontinuity is a critical decision point, or threshold, in the progression of the membrane potential towards spike initiation, at which hyperpolarization and depolarization both exert their maximal influence. The effect of this shape of SPRF is to ensure very rapid synchronization of the cell. Maximal phase shift occurs in the middle of the cycle when the phase difference is high - the postsynaptic cell either advances or delays its phase to achieve nearly immediate in-phase firing when detuning between pre- and postsynaptic cell is small. This extremely sharp midcycle transition is not observed in conventional phase-resetting relationships to weak brief inputs in these cells [37], [44], and is a consequence of the integration of the strong compound input.

The piecewise nature of the SPRF, with the phase advance contributed exclusively by gap-junctional input, and the delay component contributed exclusively by chemical inhibition, mean that these two types of connection have complementary roles in synchronization: gap junctions are necessary to entrain the firing of the postsynaptic cell to a frequency higher than its preferred frequency, while inhibitory synapses are necessary to entrain firing to a frequency lower than the preferred frequency (as seen in Figures 5 and 6). This can be seen as follows. Let *H* be the phase difference between postsynaptic and presynaptic cells (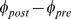). The change in *H* over one period of the input, i.e. from input *i* to input *i+1*, is: 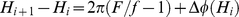. Therefore, when entrainment is achieved, 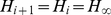, and so if *F*>*f*, then 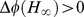, and if *F*<*f*, then 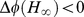.

### Using the SPRF to predict entrainment

Using the SPRF to model entrainment assumes that the effect of each stimulus in the train is the same as if it was applied in isolation. The success of the SPRF in predicting entrainment shown here demonstrates that it is at least a good approximation for this purpose, and that the arithmetic of adding effects of multiple sequential synaptic inputs behaves reasonably linearly. The SPRF assumes that the entire dynamical state of the neuron may be represented by just a single number at any time, the phase, which would imply that its dynamical state always lies on a limit cycle, along which it is kicked instantaneously forwards and backwards by the synaptic inputs. The complex dynamics of a real neuron containing a large number of different voltage-dependent conductances distributed in a complex morphology, and the strong and non-instantaneous nature of the perturbation mean that this is a considerable simplification of the reality. An indication of whether the phase approximation is reasonably valid, is to test whether there is any higher-order phase resetting, i.e. changes in the interspike interval *following* that during which the input is applied, or in subsequent intervals. When we analysed second order shifts, we found that they were sometimes detectable, but very small in relation to the first-order SPRF (See Figure S1), in line with the short memory of FS cells for input conductance fluctuations [17].

### Physiological consequences of the synaptic phase-resetting function

FS cell firing is suspected to be directly and primarily responsible for producing gamma oscillations in the neocortex [6], [7], [8]. Different fine-scale subnetworks of mutually-exciting pyramidal cells in layers 2 or 3, which are driven by specific subsets of local layer 4 inputs, appear to interact with other such subnetworks via the inhibitory interneuron network [45]. Synchronization of FS cells, therefore, may be essential for linking responses of pyramidal cells very rapidly to specific features of the synaptic input, as hypothesized to occur in sensory “binding” [2]. We have shown that the effect of conductance inputs which realistically mimic single synaptic connections on the phase of FS firing is very powerful, and is capable of entraining the postsynaptic cell even against strong noise. The strikingly sharp discontinuity between phase delay and advance in the SPRF causes a very rapid jump to nearly in-phase firing.

The relative strengths of electrical and inhibitory components can vary greatly from connection to connection [12], [13], and some pairs of FS cells connected by gap junctions can synchronize their firing, while others cannot [14]. The strengths of these components will also vary dynamically. Electrical synapses can exhibit plasticity through G protein-coupled receptor activation, intracellular calcium and phosphorylation [46], and the GABAergic connections show strong short-term depression [12], [13], [14]. These effects presumably help to shape the spatiotemporal dynamics of synchronous firing. The model that we introduce here could easily accommodate independent plasticity rules for inhibition and gap junctions, by additional rules for modifying the slopes of the corresponding regions of the SPRF. In addition to such modulation, the GABA_A_ receptor is also the target of many important neuroactive drugs, such as benzodiazepines, barbiturates and ethanol. These will be expected to influence the shape of the SPRF, and the synchronization behavior of FS cells in the gamma frequency range. The SPRF, therefore, may be a useful tool for characterizing the action of such compounds on pathological network states treated by such drugs.

Firing is considerably more variable *in vivo* than *in vitro* [47], and it is important to consider the consequences of the SPRF in strong noise. The stochastic bifurcation analysis that we carried out (Fig. 6) delineated a well-defined boundary between entraining and non-entraining frequencies, based on a qualitative change in the nature of the motion of the phase [32] (see Methods). The stronger the noise, the smaller the frequency region of stochastic entrainment – in line with intuition, noise acts to break down synchronization. The strength of the noise effect in controlling the boundary of the synchronized region is not symmetrical around *F* – thus noise can effectively shift, as well as shrink the synchronized frequency band.

In conclusion, the synaptic phase-resetting function of FS cells firing at gamma frequencies, as characterized here, is very well-suited to achieving rapid synchronization, and demonstrates complementary roles of the two types of synaptic connection in determining the frequency range of synchronization. It provides a simple yet surprisingly accurate model for predicting synchronization of these cells, and should be a useful component in network models aimed at understanding the complex spatiotemporal properties of locally-synchronized gamma-frequency firing in the cortex.

## Methods

### Slice preparation and electrophysiological recording

300 µm sagittal slices of somatosensory cortex were prepared from postnatal day 13–19 Wistar rats, using a vibratome (DSK Microslicer Zero 1, Dosaka EM, Kyoto), in chilled solution composed of (in mM): 125 NaCl, 25 NaHCO_3_, 2.5 KCl, 1.25 NaH_2_PO_4_, 2 CaCl_2_, 1 MgCl_2_, and 25 glucose, oxygenated with 95% O_2_, 5% CO_2_ gas. Slices were then held at room temperature for at least 30 minutes before recording. The tissue was visualized with an Olympus BX50WI upright microscope (Olympus UK, London) using infrared differential interference contrast videomicroscopy. During recording, slices were perfused with oxygenated solution identical to the slicing solution, at 31–35°C (8 cells analysed in detail) or 23°C (4 cells). 10 µM 2-(3-carboxypropyl)-3-amino-6-(4-methoxyphenyl)-pyridazinium bromide (SR95531; gabazine), 10 µM D-2-amino-5-phosphonopentanoic acid (AP5), and 10 µM 6-cyano-7-nitroquinoxaline-2,3-dione (CNQX) were usually added, to block chemical synaptic transmission mediated by GABA_A_, N-methyl-D-aspartic acid (NMDA),and α-amino-3-hydroxy-5-methyl-4-isoxazole proprionic acid (AMPA) receptors, respectively. Whole-cell recordings were made from the somas of nonpyramidal neurons in cortical layers 2/3, 4, and 5. Cells identified as FS neurons had a mean input resistance of 202±87 MΩ (*n* = 12). Data from 10 fast-spiking neurons (taken from 8 animals) were used for analysis, with a further 12 cells showing consistent results, but which were not complete enough for analysis. The number of synaptic phase-resetting functions with different parameters of the conductance perturbations (see below) which could be constructed for each cell was limited by the lifetime of the recording, typically 20 to 40 minutes.

Patch pipettes of 3–5 MΩ resistance were pulled from borosilicate capillary glass and filled with an intracellular solution containing (in mM): 105 K-gluconate, 30 KCl, 10 HEPES, 10 phosphocreatine, 4 ATP, 4 MgCl_2_, and 0.3 GTP, adjusted to pH 7.3 with KOH. Current-clamp recordings were performed using an Axon Multiclamp 700A or in a few cases, an Axopatch 200A amplifier (Axon Instruments, Foster City, CA). Membrane potentials were corrected for nulling of the liquid junction potential before seal formation. Signals were filtered with a four-pole low-pass Bessel filter at −3dB cutoff frequency of 5 kHz, sampled at 20 kHz, and recorded with custom software written in MATLAB (The Mathworks, Natick, MA).

### Conductance injection

Recorded neurons were stimulated using artificial conductance injection [25], [26], [48]. An effective conductance is inserted in the recorded cell by injecting a current *I* according to Ohm's law, *I* = *g*(*V*−*E*_rev_), where *g* is the conductance, *V* is the membrane potential of the cell, and *E*_rev_ is the reversal potential of the conductance. A conductance injection amplifier [49] or digital signal processing system (SM-1 or SM-2, Cambridge Conductance, Cambridge, UK) [50] with response times of less than 200 ns or 10 µs respectively, were used to calculate and produce the current command signal in real time for the current-clamp amplifier.

Steady trains of action potentials at gamma frequencies were elicited by steps of AMPA-receptor like ohmic conductance, reversing at 0 mV, to which perturbing conductances were added as follows. Stimuli that mimicked action potentials filtered through electrical synapses were generated. An action potential (AP) waveform was produced using a conductance-based model of an FS cell, identical to that of [41], except that the leak conductance was reduced to better fit the stimulus-response curves of actual FS cells (see **Fast-spiking cell conductance-based model (section below)**

This AP waveform was then used as the time-varying *E_rev_* signal for a constant conductance *g_e_*, representing the electrical synapse. The conductance of a unitary synaptic GABA event was modelled as a difference of exponentials 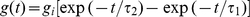, where 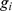 is the scaling amplitude of the inhibitory conductance, and 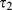 was 7 ms, and 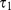 was 0.5 ms. In compound stimuli, the start of the GABA event was delayed by 3 ms from the start of the simulated action potential to represent synaptic latency. The reversal potential *E*_GABA_ was usually set to −55 mV [29].

### Data analysis

Spike times were determined as the times of positive-going threshold crossings of the membrane potential at a threshold set at 10 mV below the peak of action potentials. The phase at which a stimulus was applied was calculated from the time elapsed from the preceding spike, relative to the unperturbed firing period. Variability of phase was characterized by the phase order parameter, or synchrony 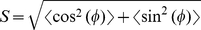, which varied between 0 (phases distributed uniformly between 0 and 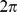) and 1 (phases all identical). The change in phase (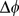) caused by a stimulus was calculated as follows. Let 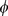 be the phase reached at the moment of perturbation, 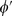 the phase immediately after, 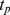 the time after the *previous* spike at which the perturbation is applied, 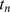 the time elapsed after the perturbation before the *next* spike, and 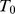 the average interspike interval. Then 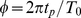, 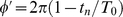 and 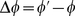.

### Fitting and simulations

The synaptic phase-resetting function (SPRF, see Fig. 2) was approximated by the piecewise linear relationship:
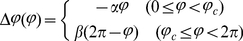
(1)where conductance values are in nS, -*α* is the slope in the phase advance section, -*β* is the slope of the phase delay section, and 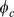 is the breakpoint. SPRFs were fitted to experiments by least-squares, and using Grubbs' test for outliers, to delete occasional outlying points (in most cases none, but no more than three per SPRF).

Entrainment of periodic spiking to periodic stimulation was simulated by the noisy map describing the evolution of the phase from stimulus *n* to stimulus *n+1*:
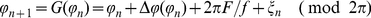
(2)where *f* is the stimulus angular frequency, *F* is the unperturbed (natural) angular frequency of the cell, and 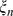 is a Gaussian-distributed noise term, with variance 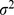. The biophysical simulations of Fig. 7 were carried out using the model specified by [41], modified slightly as described above (see **Conductance injection**).

### Bifurcation analysis

Bifurcation points, where 1∶1 entrained fixed points of the map given by Eq. 2 appear, were solved for directly. To determine the points of *stochastic* bifurcation, we used the definition of [32]. The stochastic map of the phase between successive stimuli on a unit circle *S* is represented by a Markov operator *p* on the phase distribution, where 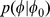 is the conditional probability density function of the phase at stimulus *i+1*, given a phase of 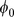 at stimulus *i*.
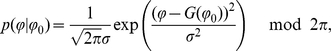
and the distribution of phase 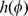 advances from stimulus *n* to stimulus *n+1* according to:
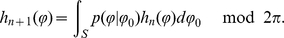

*p* is approximated by a stochastic transition matrix, and the onset of stochastic entrainment is defined by the point where the second eigenvalue of this stochastic transition matrix changes from real to complex. This definition of a stochastic bifurcation coincides with the deterministic case as the noise level approaches zero, is clearly defined even when the steady-state phase distribution hardly changes, and incorporates the dynamics of the phase: the first eigenfunction gives the stationary or invariant distribution of the phase, while the second eigenfunction can be thought of as forming the principal component of the average time course of relaxations from an initial random phase distribution.

### Fast-spiking cell conductance-based model

A model of fast-spiking cell membrane potential (*V*) dynamics was used (as above for generating action potentials for gap-junctional stimulation) which was slightly modified, with a different leak conductance, from that specified in Erisir et al., 1999 [41] (also correcting typographical errors in the published description of the model). Sodium (Na), Kv1 (K1) and Kv3 type potassium and static leak (L) conductances were used in a single electrical compartment of capacitance C, as follows (units of mV for voltage, ms^−1^ for rates):
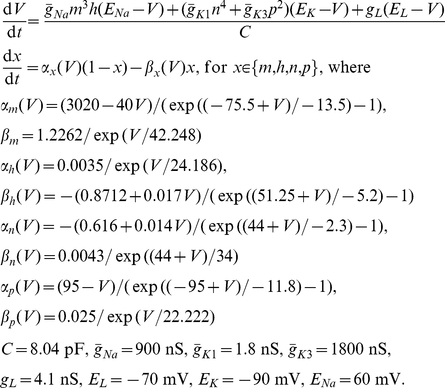
Exactly the same conductance stimuli were applied to the model as to cells experimentally (see **Conductance injection** section above).

## Supporting Information

Figure S1 An example of the lack of phase shift in the cycle following that in which a strong perturbation is applied (second-order resetting). *F* = 61 Hz, *g_e_* = 0.4 nS, *g_i_* = 2 nS. Dashed lines indicate expected standard deviation if there is no second order effect.
(0.09 MB TIF)
