# Supplementary figures and images for: Synchronization of Firing in Cortical Fast-Spiking Interneurons at Gamma Frequencies: A Phase-Resetting Analysis
Source: PLoS Comput Biol. 2010 Sep 30;6(9):e1000951. doi: 10.1371/journal.pcbi.1000951 (PMC2947988; doi:10.1371/journal.pcbi.1000951)

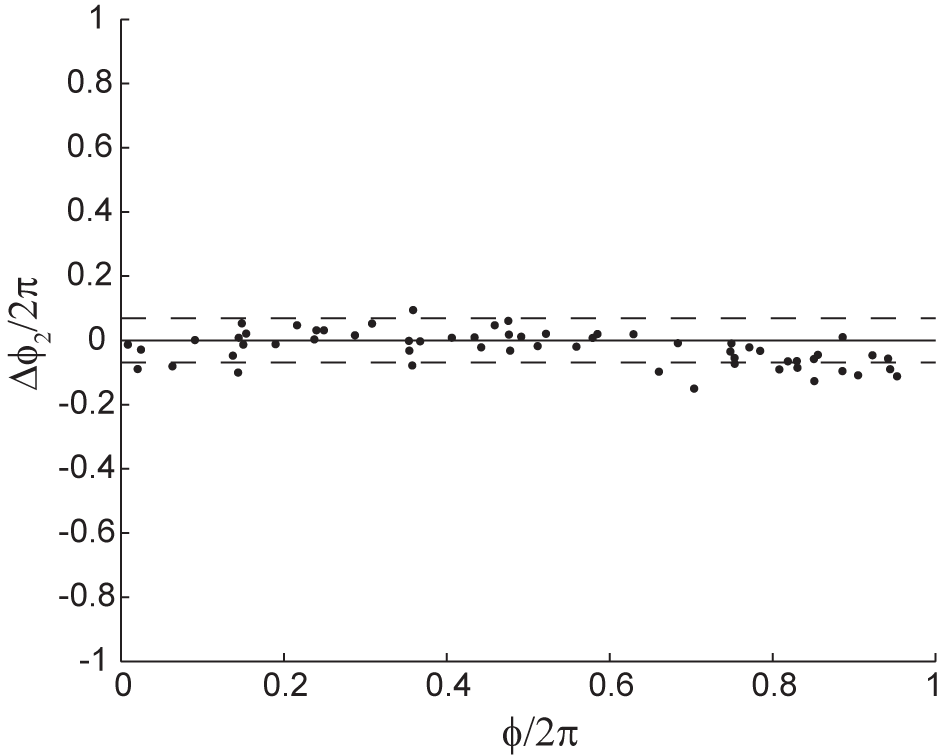

Supplement: Figure S1 — An example of the lack of phase shift in the cycle following that in which a strong perturbation is applied (second-order resetting). F = 61 Hz, ge = 0.4 nS, gi = 2 nS. Dashed lines indicate expected standard deviation if there is no second order effect. (0.09 MB TIF) [file pcbi.1000951.s001.tif]
